# Handover Parameters Optimisation Techniques in 5G Networks

**DOI:** 10.3390/s21155202

**Published:** 2021-07-31

**Authors:** Wasan Kadhim Saad, Ibraheem Shayea, Bashar J. Hamza, Hafizal Mohamad, Yousef Ibrahim Daradkeh, Waheb A. Jabbar

**Affiliations:** 1 Engineering Technical College-Najaf, Al-Furat Al-Awsat Technical University (ATU), Najaf 31001, Iraq; coj.bash@atu.edu.iq; 2 Electronics and Communication Engineering Department, Faculty of Electrical and Electronics Engineering, Istanbul Technical University (ITU), Istanbul 34467, Turkey; ibr.shayea@gmail.com; 3 Faculty of Engineering and Built Environment, Universiti Sains Islam Malaysia, Bandar Baru Nilai, Nilai 71800, Malaysia; hafizal@usim.edu.my; 4 Department of Computer Engineering and Networks, College of Engineering at Wadi Addawasir, Prince Sattam Bin Abdulaziz University, Al Kharj 11991, Saudi Arabia; y.daradkeh@psau.edu.sa; 5 Faculty of Electrical & Electronics Engineering Technology, Universiti Malaysia Pahang, Pekan 26600, Malaysia; waheb@ieee.org; 6 Center for Software Development & Integrated Computing, Universiti Malaysia Pahang, Gambang 26300, Malaysia

**Keywords:** load balancing (LB), handover (HO), handover control parameters (HCP), handover parameters optimisation (HPO), fifth generation (5G), sixth generation (6G) networks

## Abstract

The massive growth of mobile users will spread to significant numbers of small cells for the Fifth Generation (5G) mobile network, which will overlap the fourth generation (4G) network. A tremendous increase in handover (HO) scenarios and HO rates will occur. Ensuring stable and reliable connection through the mobility of user equipment (UE) will become a major problem in future mobile networks. This problem will be magnified with the use of suboptimal handover control parameter (HCP) settings, which can be configured manually or automatically. Therefore, the aim of this study is to investigate the impact of different HCP settings on the performance of 5G network. Several system scenarios are proposed and investigated based on different HCP settings and mobile speed scenarios. The different mobile speeds are expected to demonstrate the influence of many proposed system scenarios on 5G network execution. We conducted simulations utilizing MATLAB software and its related tools. Evaluation comparisons were performed in terms of handover probability (HOP), ping-pong handover probability (PPHP) and outage probability (OP). The 5G network framework has been employed to evaluate the proposed system scenarios used. The simulation results reveal that there is a trade-off in the results obtained from various systems. The use of lower HCP settings provides noticeable enhancements compared to higher HCP settings in terms of OP. Simultaneously, the use of lower HCP settings provides noticeable drawbacks compared to higher HCP settings in terms of high PPHP for all scenarios of mobile speed. The simulation results show that medium HCP settings may be the acceptable solution if one of these systems is applied. This study emphasises the application of automatic self-optimisation (ASO) functions as the best solution that considers user experience.

## 1. Introduction

The explosive growth of mobile communications, the diversity of networks, and three-dimensional (3D) mobile communications (e.g., drones) will radically increase mobile data demands, in which servicing will require a large number of UEs for deploying huge amounts of small and interfering BSs [1,2,3]. With the rapid growth of the Internet of Things (IoT), the 3rd Generation Partnership Project (3GPP) proposed a new wireless network generation (5G) to address the numerous challenges faced by existing networks. However, an equally important and additional aspect discussed by 3GPP through the efforts of ongoing standardisation is the aspect of mobility in 5G networks. To become the new wireless standard to exist worldwide, 5G must allow for unrestricted user mobility while effectively managing itself. The methods, as discussed in [4], aim to provide management efficiency by applying techniques that emphasise requests based on mobility management (MM). An essential component of MM is HO management, since HOs allow users to switch the network anchor point while maintaining service continuity through mobility events in existing cellular networks. Thus, effective HO management will be vital due to the heterogeneous and extremely dense nature of 5G networks [5,6,7,8,9,10,11,12,13].

The access of mobile phone users and the resulting traffic load in cellular networks are variable over time, frequently unbalanced and random, making cell loads in the system unequal. In some cells, excessive amounts of UEs are available but overloaded; while, in other cells, fewer UEs are present, and their resources are not fully utilised. The inefficient use of resources can be mitigated through optimised administration as well as network development. The current network planning strategies are far from completely resolved, due to issues such as load balancing (LB) in long-term evolution (LTE) systems [8,14,15,16]. Generally, converting some UEs at the boundaries of overlapping or adjacent cells from more crowded cells to less crowded cells represent a possible approach for LB; this is often referred to as HO or handoff. By changing the Evolved Node B (eNB) units assigned to UE, the load is balanced, and the system performance is improved at the expense of system overheads, generated by HOs. The HO procedure consumes significant system resources, and the intended UE may experience substantial system delays and performance deterioration. Therefore, HO should not randomly occur [17,18]. Additionally, the use of mm waves in 5G technology is the dominant factor that impacts mobility [19,20]. This is due to higher path loss when using mm-wave frequency bands, thereby decreasing cell coverage. HO probability will significantly increase, leading to an upsurge in the number of mobility issues, such as high HOF, PPHP impact and OP.

The Handover Parameters Optimisation (HPO) is an important function of the self-optimisation network (SON). It was introduced by 3GPP for solving the mobility issues in 4G and 5G mobile phone networks [21,22,23,24]. Several functions are offered by SON, such as mobility robustness optimisation (MRO) and load balancing optimisation (LBO). Both functions lead to necessary optimisations to achieve various goals throughout the mobility of the utilizer and aims to dynamically enhance HCP values to confront numerous HO problems. MRO was initially proposed in the LTE-Advanced (LTE-A) as part of SON, where it sets HCPs, such as handover margin (HOM) and time to trigger (TTT), to maintain communication links throughout user movements with a minimum number of overlapping operators [25].

**Figure 1:**
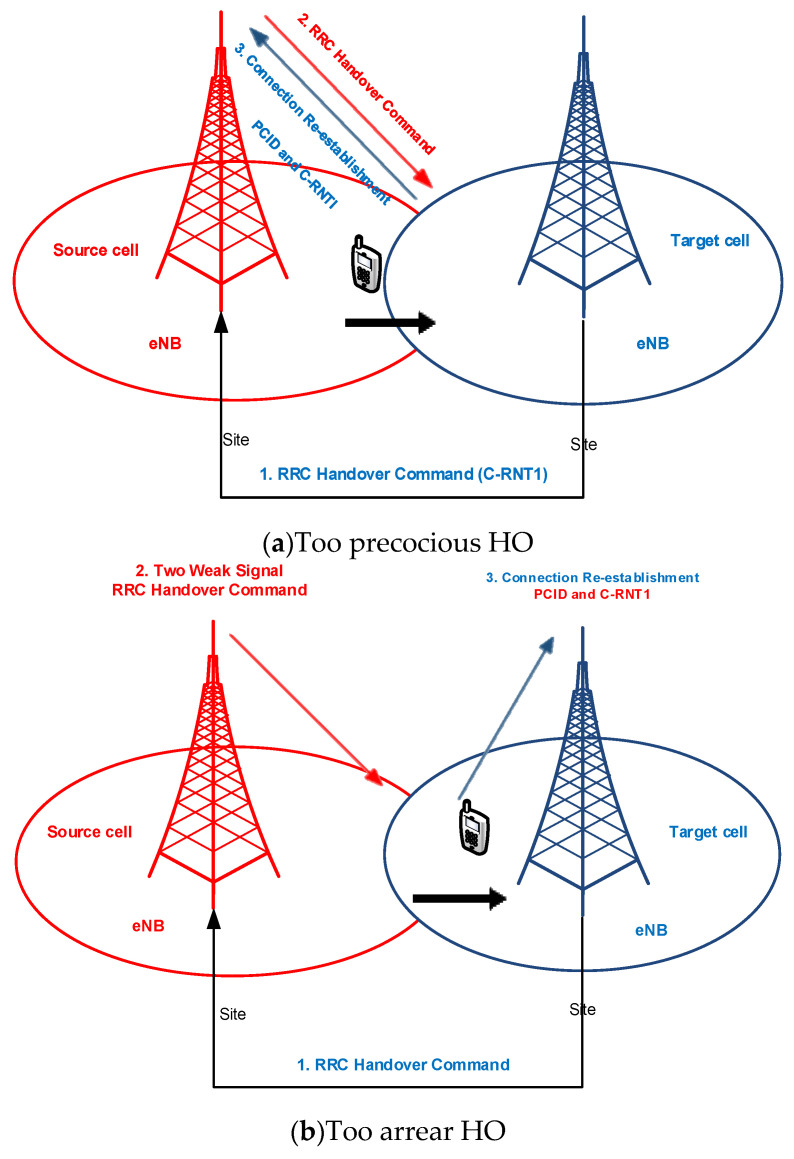
HO problems because of settings of suboptimal HCP [34].

The function of MRO automatically adjusts the values of HCP to preserve the quality of the system. It also automatically optimises HCPs with minimum human overlap. Through adjusting these parameters to appropriate values by the movements of the utilizer within the coverage of the cell, PPHP rates and handover failure (HOF) are adequately reduced, thus improving the quality of service (QoS) [26,27]. HPO functionality was presented as a primary merit in deploying 4G and 5G networks. Its key goal is to automatically adjust HCP settings to maintain the quality of the network. The specific goal of HPO is to detect and correct the impact of both PPHP and OP because of mobility. In another sense, the HPO algorithm adaptively adjusts the settings of HCP when OP or PPHP is detected because of HO is too precocious and HO is too arrear, as shown in Figure 1. It can also be due to the ineffective usage of the resources of system, caused through unnecessary handover (UHO).

One of the main methods for improving the performance of 5G network mobility is optimising HCP settings. If HCPs are set to static settings, continuous connection will be adversely influenced, particularly when UE speed is remarkably high. Therefore, HCP settings must be appropriately modified to resolve this deficiency. However, manual modification will complicate the maintenance and management of the system. As a result, the HPO function has been submitted by 3GPP as the primary feature in 4G and 5G network deployment [28,29]. This function automatically estimates the occasion settings of HCP, depending on the conditions of the instantaneous network. Various studies were accomplished to handle this insufficiency [11,30,31,32,33].

In the literature, several algorithms exist to optimally calculate HO parameters, such as TTT and hysteresis value. The algorithms in [35,36] are methods for overcoming the influence of the ping-pong effect due to HO. HO optimisation between femto and macro BS (by exploiting the information of UE, such as velocity, RSSI, etc.) was also discussed in [37]. Several utilised algorithms have demonstrated ineffective input parameters in their design, resulting in the inaccurate estimation of HCP settings. Currently, mobility with high requirements within 5G networks (such as mm waves and lower latency) has led to the requirement of HPO algorithms that are further progressed. It has become a major development requirement to successfully process the mobility problems found in 5G networks. The available algorithms in the literature [11,30,31,32,33] provide effective optimisation of HCP settings; however, there is no perfect solution, since some suggested algorithms only adjust HCP settings depending on the single parameter (e.g., distance or speed). While many affecting factors must be considered to estimate appropriate HCP settings (e.g., distance, interference, channel state, resource availability, noise and UE speed), these configurations are approximated from a single factor perspective will only cause insufficient HCP settings. Some of these algorithms, such as the Adaptive Handover Algorithm (AHA), are dependent on the distance, speed and Fuzzy Control Algorithm (FCA) [38,39,40] where the FCA only sets the HOM level, and the TTT is set to a constant value. This failure diminishes the key aim of the HPO, task all characterised algorithms and optimise per cell, excluding AHA. This may allow for some UEs to execute the HO for other cells, while they do not need to perform HO at that time; thus, unnecessary HOP will increase due to suboptimal HCP settings.

The objective of this paper is to reduce the OP and PPHP occurrences through HO processes while adjusting HCP settings. Several system scenarios have been proposed, such as fixed HOM values and fixed TTT intervals, based on various HCP settings and different mobility speed scenarios, to clarify the effect of these scenarios on 5G network performance. The framework of the 5G network was applied to evaluate the proposed systems scenarios, utilised by comparing the HOP, PPHP and OP with different mobile speeds. The proposed scenarios were then investigated and compared according to the simulation study through utilizing MATLAB 2020a software.

The key contribution of this paper is investigating the effect of various HCP settings on the performance of 5G networks in terms of PPHP and OP. Several system scenarios are proposed, according to various HCP settings with different mobility speed scenarios. The proposed systems in this paper provide noticeable differences in system performance with the use of lower HCP settings, compared to higher HCP settings. The results indicate that medium HCP settings may be a better solution when utilising one of these systems. The consideration of automatic optimisation is another excellent solution that shall be focused on in future investigations and developments.

In summary, this work provides the following listed contributions:

- The impacts of various HCP settings on the system performance of the 5G network are studied based on three key performance metrics: HOP, PPHP and OP.
- Various HCP system settings, HOM and TTT are proposed and investigated for the 5G network with various scenarios for the speed of mobile.
- The suggested systems are validated to ensure their efficiency in the 5G network.

The rest of this paper is organised as follows: Section 2 presents the related works of this study. Section 3 briefly discusses the main HO performance evaluation metrics. Section 4 highlights the system model and the proposed solution of the simulation scenario used in this paper. Section 5 discusses the simulation and performance evaluation analysis. Finally, Section 6 presents the conclusions of this paper.

## 2. Related Works

A major challenge in wireless networks is the use of numerous algorithms, suggested in the literature for optimising HCP settings [41,42,43,44,45,46,47,48]. Several methodologies have applied these algorithms and examined them in different environments. The authors in [41] proposed the machine learning and data mining (MLDM) technique to optimise HO parameters that have been evaluated in the long-term evolution (LTE) system within the a building environment. In addition, a high-mobility SON function has been offered in [42] to shorten the multi-layer time that performs the optimisation process in the LTE system. It estimates the behaviours of user mobility according to data measurements, previously collected through utilizers. Furthermore, the authors in [44] presented an algorithm to adaptively adjust HOM, based on the user’s position in the cell, whereby, the HOM further decreases the closer the utilizer is to the edge of the cell. The researchers in [43] presented an adaptive algorithm that specifies various HOM values and load balancing (LB) to every UE in HetNets. The decision of HO in this suggested algorithm is based on the signal to interference noise ratio (SINR) instead of the strength index of the received signal, which is subsequently utilised to compute the HOM actual level. The authors in [44] also introduced an Enhanced Mobility Status Estimate (EMSE) for optimising HCPs according to HO types and user velocity by estimating the state of mobility. In this paper, the authors updated TTT by solely depending on user speed with limited TTT refresh values. This method did not completely optimise the performance of HO because the gap between the refresh values was too large, and the three fixed values were only chosen according to the UE speed. The authors in [45,46] discussed several executions of LTE HO frequencies, while the researchers in [47,48] considered the optimisation of the inter-system HO parameter.

Various studies have suggested several algorithms for solving and addressing HO problems. The authors in [49] described the strategy of HO decision and the estimation scheme of the mobility state to avoid service failure and unnecessary HOs (UHOs) in HetNets. The suggested model uses the HOs number, as well as the measurements of residence time, to appreciate the speed of UEs. Simulation results indicated that the suggested model’s mobility state estimation reduces service failures and UHO number; however, its execution in relation to other performance metrics of HO were not discussed, such as Outage Probability (OP), PPHP and delay.

The authors in [32,33] suggested an HO optimisation technique, according to the weighted function of the conveyor assembly. The suggested algorithm automatically modifies the HOM values, based on three functions: speed, traffic load and SINR. The simulation results revealed that the suggested algorithm would boosts execution of the system in terms of OP and spectral efficiency at the edge of the cell.

The authors in [50] proposed a method to compare the intersections of cell boundaries and the implementation of HO to optimise the performance of the overall network.

The researchers in [51] adopted a model for HOM optimisation according to fuzzy logic for HetNets, where the fuzzy logic consisting of two inputs, the LB index and the call drop rate, both of which consist of HOM adaptations for small and macro cells.

The authors in [11,30,52] proposed several algorithms for investigating and evaluating the mobility management problem in various mobile phone velocity scenarios. Three types of HO have been considered for conditioning HCPs: very late, very early and HO for wrong cells. The simulation results revealed that the modified HCP providers reduce the HOP, OP and HPPP rates.

However, the proposed HO-SON algorithms in the aforementioned papers were found to be inefficient in estimating optimum HCP settings. Although these algorithms contribute to enhancing the performance of HO, but it is neither strong nor ideal in choosing the occasional values of HCP in the 5G system. The current algorithms are inadequate for different reasons. One of the key reasons is that most of these algorithms were developed for 4G technology, which have various requirements and specifications compared to 5G technology. Further investigations are needed to develop the existing algorithms used in previous cellular networks to be effectively executed in 5G networks with different scenarios of mobility and deployment. Another reason is the HCP types considered for optimisation; several current algorithms do not optimise all HCP settings. For instance, algorithms in [39,53,54] only optimise one HCP (i.e., HOM) which may cause an increase in HO. Using a static TTT could lead to another HO problem which the Handover Parameter Optimisation (HPO) aims to process. Thus, the HPO algorithms must be developed with high efficiency and should be validated for 5G networks.

In [55], the threshold approach to the HO procedure has been incorporated into the multi-criteria decision-making (MCDM) process to select the Radio Access Technologies (RATs). The available RATs rankings are ordered through different MCDM algorithms and, depending on the specific threshold of HO, where the decision of whether or not to perform the process of HO is made. The suggested method works to improve the performance of the system, as well as to reduce HO by 13.14%, 19.35% and 8.62% of the RAT amendments for the technique for order preferences by similarity to the ideal solution (TOPSIS), preference ranking organization method for enrichment evaluation (PROMETHEE) and simple additive weighting (SAW) algorithms, respectively.

In [7], the algorithm for speed-based self-optimization was proposed to modify the HCPs in 4G/5G networks. The suggested algorithm uses the user’s received velocity and power to modify the TTT and HOM as the user navigates in the network. The simulation results show that the suggested algorithm outperforms the other existing algorithms by a rate of more than 70% for all measures of HO performance.

In [56], the authors proposed a new selection mechanism of context-aware radio access technology (CRAT), which examines user and network contexts in selecting the appropriate RAT for a service. The proposed CRAT performance was tested by using two various scenarios through a smart city environment, namely urban city and shopping centre scenarios by changing the environment parameters for measuring the performance of the proposed mechanism in a near-realistic situation. The results demonstrated that CRAT can help to enhance the utilizer experience through a smart city environment, where it outperforms the traditional A2A4 approach to choose the RATs in terms of number of HOs, throughput, packet delivery ratio and average network delay. Generally, Table 1 briefly summarizes the HO optimisation approaches and displays the utilized data model type and how to take HO decisions.

Although several studies that focused on HCPs have been conducted in the literature, they are mostly focused on 3G and 4G networks. This means that there is a need for further investigation in 5G networks, with various system scenarios and settings. Moreover, there is no comprehensive study that has considered all of the key performance indicators (KPIs). Thus, in this paper, the impact of different HCP settings has been considered for 5G network performance through various proposed system scenarios with different mobility speeds, all of which are modified depending on the PPHP and OP conducted in the measurement period. Meanwhile, system performance considered various KPIs. The simulation results reveal that the proposed system provides notable improvements with the use of lower HCP settings in terms of OP compared to higher HCP settings. The noticeable drawback of increased PPHP for different mobile speed scenarios is also present. The results prove that medium HCP settings may be the best solution to consider when using one of these systems.

## 3. The Key HO Performance Evaluation Metrics

Many performance indicators or main performance metrics (MPMs) are frequently defined in wireless networks to determine Quality of Service (QoS). To analyse the proposed HO preparation and signals of failure, HOP, PPHP and OP are the metrics used for evaluation. Employing these metrics is standard practice for assessing new HO strategies. Thus, the proposed algorithm fulfils this criterium and the indicators are compared with previous algorithms using three key MPMs, as follows:

**HOP:** This is the probability of links exchanged between the source and the target eNBs, where how HO repeatedly occurs between the source and target eNBs is measured. In other words, it is basically handing probability through the served UE from the source to the target eNBs when the quality of the source signal becomes worse than the strength of the target signal by the level of HOM. Thus, HOP can be translated into the HOs average number per call across all UEs served for increasing the accuracy of performance appraisal. The average HOP is computed per simulation period across all UEs served in the network, hence, the HOs average number per UE $\left(\bar{HOP}\right)$ can be mathematically shown as follows [7]:

(1) $$\bar{HOP}=\frac{\sum_{i=1}^{M_{UE_{s}}}HOP(i)}{M_{UE_{s}}}$$

where $M_{UE_{s}}$ is the total number of served *UEs* in the whole simulation through the network, and HOP(i) is the HOP for *UEi*.

**PPHP**: This is an important measure in HO studies since it calculates the number of UHOs made between two adjacent cells. On other words, the PPHP is the UHO that may occur due to sub-optimal HCP settings. The HO will experience the impact of ping pong if UE-i leaves the eNB-A service to the eNB-B target and then returns to the eNB-A service in a period below the critical period $\left(T_{c}\right)$. This represents the critical time required for measuring the UHO between neighbouring cells, which is supposed to be 2 s [59]. Thus, HPPP can be measured when HO occurs, depending on the following equation:

(2) $$PPHP=P\left[\left(T_{l}-T_{r}\right)\leq T_{c}\right]$$

where the interval time represents the difference between $T_{l}$ (the time at which the UE leaves the serving eNB-A) and $T_{r}$ (the time at which the UE returns to the same serving eNB-A). Thus, the HO is recorded as a ping-pong handover (PPH) if the UE returns to the same serving eNB-A and the interval time is below $T_{c}$. The number of PPHs is recorded for every UE, while the average PPHP through all service UEs is recorded in each simulation period t to increase performance evaluation accuracy. Therefore, the average HPPP $\left(\bar{PPHP}\right)$ for UE through the simulation period *t* can be represented as follows [7]:

(3) $$\bar{PPHP}=\frac{M_{PPH}}{M_{F}+M_{PPH}+M_{NPPH}}$$

where $M_{PPH}$ is the total number of *PPH* throughout the entire system, and the total number of requested HOs represents the sum of failed HOs $\left(M_{F}\right)$,and non-*PPH* $\left(M_{NPPH}\right)$ numbers, respectively.

**OP**: It is defined as the percentage of the area within the cell that does not meet the minimum power (Pmin) requirements. It is the probability that the immediately received SINR (η) level is less than a certain threshold level. The threshold level (*η*th) is the minimum SINR level where performance would be unacceptable below it. Thus, the *OP* for mobile communication systems is mathematically expressed as follows [60,61]:

(4) OP=P[η≺ηth]=1−P[η≻ηth]

The *OP* is recorded when the serving SINR of the UEi is less than a certain threshold level through the simulation cycle t, where the average *OP* for all UEs is computed through each simulation cycle to increase the accuracy of results. Thus, the average *OP* $\left(\bar{OP}\right)$ can be simplified from Equation (4), as follows [7]:

(5) $$\bar{OP}=\frac{\sum_{i=1}^{M}1-P\left[\eta ≻\eta \mathrm{th}\right]}{M_{UE_{s}}}$$

The sub-optimal settings of HCP can be determined statically or estimated automatically, similar to *PPHP*, with various directions. Generally, the *OP* occurs in the static state when the settings of HCP are manually selected at extreme levels. The *OP* occurs in the automatic state if inconvenient HCP settings are automatically estimated through the HPSO algorithm. Either way, the semi-ideal HCP settings usually cause OP to occur if the settings of HCP are at extreme levels. This causes HO lagging, which may later lead to an increase in the OP in some positions, particularly for mobile utilizers who are at the edges of cell or those who move at high mobile velocities. This will consequently cause an increase in network resource wastage, which will reduce the performance of the network. Thus, it is necessary to reduce the OP as much as possible to conserve network resources.

## 4. The Simulation Scenario and System Model

The optimised HCPs represent the HCPs that are considered to be automatically estimated (optimised) according to a particular case. The suggested algorithm demonstrates the dynamic estimation of HCP settings considered in this work, which includes the HOM values and TTT intervals, respectively. This dramatically contributes to the estimation of the most suitable HCPs for every UE, according to their independent experiences. The negative effect on other UEs, which do not require any changes in HCP settings, can also be avoided. This will reduce the PPHP and OP, respectively, which will lead to tremendous improvements in providing more stable communications throughout UE mobility.

The HOM represents one of the key parameters that is employed to control the HO decision. Low or high HOM settings may lead to high PPHP, high OP or similar problems that are unsatisfactory in wireless systems. Further adjusting the cell’s HOM settings, where all users within the cell will utilise the same HOM, can also create one of these problems. This situation becomes even more important in 5G networks and beyond because of very small coverage from the application of mm waves. Accordingly, there is a needed to the automated mode to separately estimate HOM settings for every utilizer. However, HOM modification is very sensitive and must be implemented with care. In this work, several system scenarios are proposed according to various HCP settings with different mobility speed scenarios to dynamically and separately estimate the incidental HOM settings for every utilizer based on this presumption. The proposed systems contain two parts: the first part is the HOM threshold level, which is defined as the static value, and the second part is the dynamic and continuous amendment for every individual utilizer. Therefore, the total HOM level can be automatically estimated by the sum of the fixed threshold and the adjusted portion, which is mathematically shown, as follows:

(6) $$HOM=0.5*\left(H_{\max}-H_{\min}\right)+M_{ap}$$

where the fixed threshold is the fixed value calculated as an average *HOM* setting, representing the half difference between the maximum and minimum HOM values $\left(H_{\max},H_{\min}\right)$. This was assumed to be 10 dB and 0 dB, respectively [62,63]. The $M_{ap}$ is the adjusted part of *HOM*.

The TTT interval is another important HCP setting, and takes the range defined by 3GPP in [64]. The set TTT intervals change from 0 to 5.12 s. Nevertheless, the higher or lower TTT intervals may result in high PPHP or high OP, respectively. Therefore, the best solution would be automatic tuning, according to user and network performances. However, adjusting the TTT for all users in the cell throughout the entire system might pose critical problems for some because they have a variety of different experiences. Some utilizers may have good experiences at the boundaries of cell, while others may have bad experiences. Individually adjusting the TTT for each user would be a good solution; however, the adjustment must be carefully conducted. We further suggest adjusting the TTT up or down by increasing or decreasing the TTT threshold with a fixed period.

**Figure 2:**
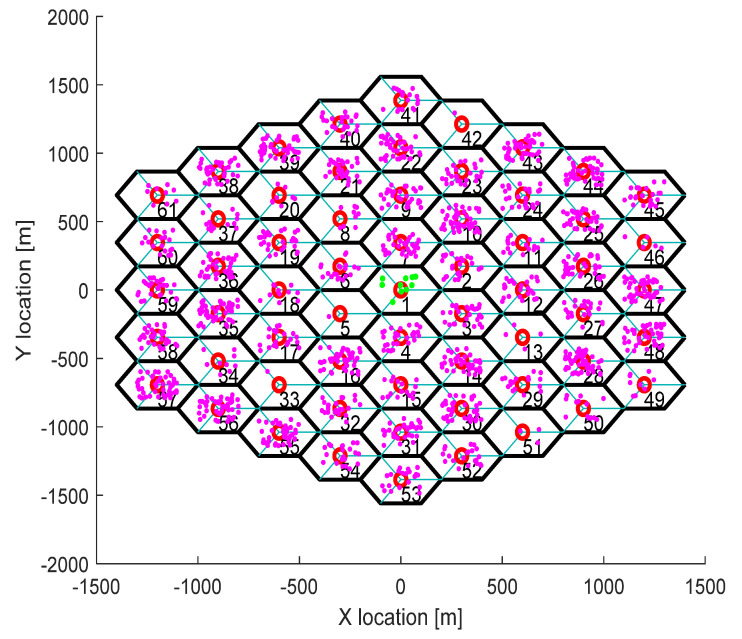
Network deployment of various hexagonal cells, each cell containing three sectors.

The simulation model in this work has been developed to simulate a real 5G network. The network has been designed based on the specifications of LTE-Advanced Pro 3GPP Rel. 16, as shown by 3GPP in [65,66], with the presumption that the network environment will be micro cells, Urban areas and 5G Rel. 16 System. Regarding the network deployment design, each hexagonal cell, constructed with a space between sites, has a cell radius R (m) and one eNB located in its centre. Every hexagonal cell contains three sector antennas, which equip every user with an omni-directional antenna to connect to the service network. However, the number of hexagonal cells can be automatically increased according to the simulation time period specified in the simulation. Figure 2 presents an example of the 5G network deployment scenario used in this work with hexagonal cells, each containing three sectors, where the cross shape represents the UE, while the triangular shape represents the eNB, respectively [33].

To represent the environment of the real 5G network, initially, a number of mobile utilizers were created with random coordinates inside the hexagonal boundaries of each cell. The 200 randomly distributed utilizers are generated throughout every hexagonal cell. The utilizers’ number periodically and randomly changed in each cell during the simulation cycles. This is means that the load traffic for every eNB was periodically and automatically changed to represent this real environment. This is taken into account in the simulation model, developed in order to simulate the arbitrary generation of load traffic during the simulation and to fully enable the acceptable control functions in the target cell during user mobility.

**Figure 3:**
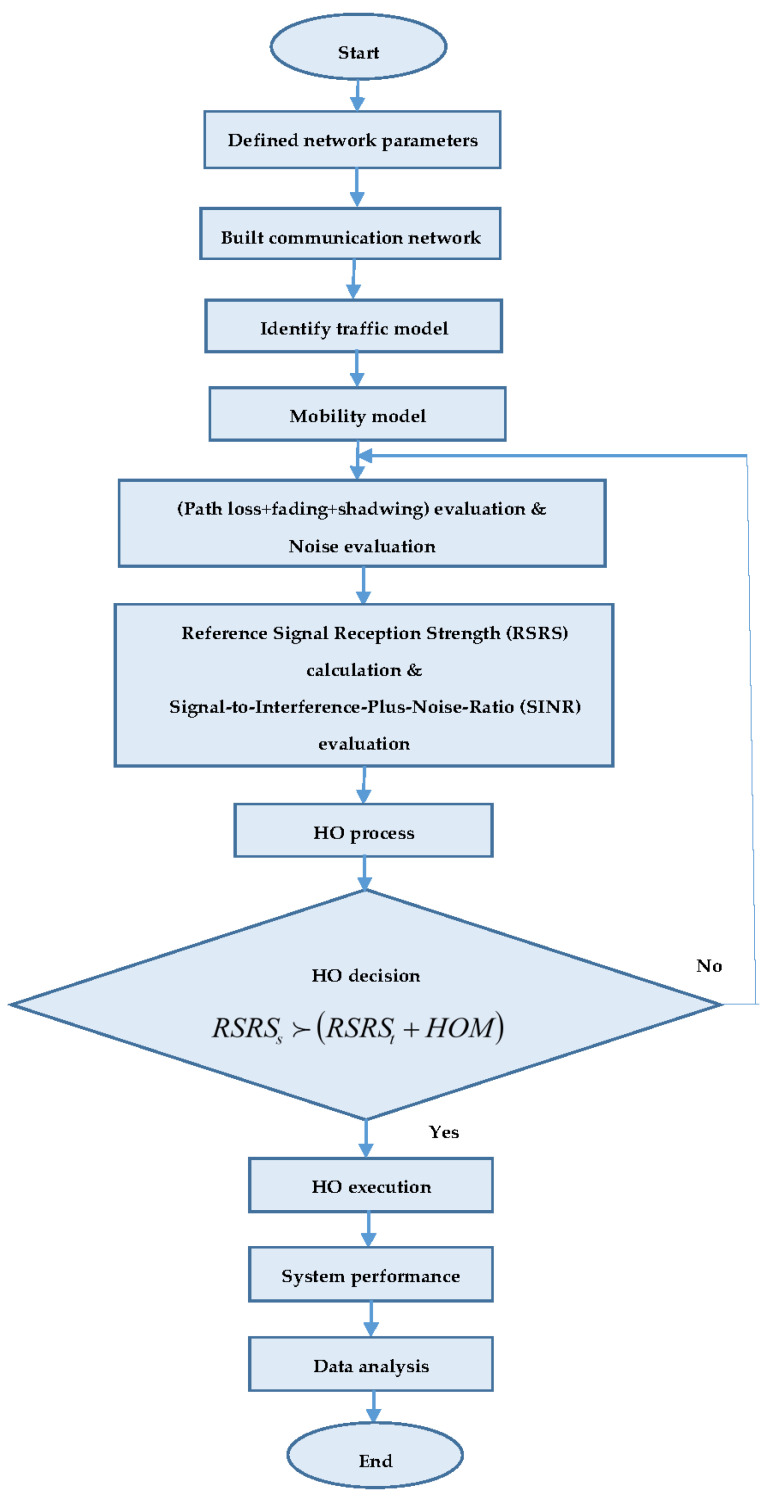
Flowchart of the proposed simulation model.

The proposed algorithm has been evaluated and validated through the simulations using the 5G network. The average values taken from 15 users represent all results considered in the measurements throughout this work. The 15 users were randomly generated within cell number 1 in the first simulation period. Initially, every user had various random coordinates in the cell. Each utilizer had a varied path that was parallel to another utilizer’s measure, since user mobility is directed in one direction. This means that all utilizers will move parallel to each other user in one direction. The directional mobility model has been suggested for all mobile device users measured across the network that are permitted to move in one direction only. This will increase the accuracy of the results since performance is measured independently for every utilizer in each simulation period. This is accomplished in 5 s through their mobility inside the cells, matching the movement distance with the periodic interval. Then, the average value was taken for all utilizers, which were measured in each simulation cycle. Therefore, the results for the average values of HOP, PPHP and OP were computed in each simulation cycle. This represents the average values of all 15 users, since they move in parallel through various pathways within cells. The measurement procedure has been accomplished to illustrate wireless network performance according to the proposed HPSO algorithm. In this work, it is assumed that the initial values for HOM and TTT for the applied HPO algorithm are 2 dB and 100 ms, respectively. The simulation began according to parameter settings, shown in Table 2 and in the flowchart in Figure 3.

The required parameters of the network were first defined and then the entire simulation network environment was built, followed by the mobility model. The directions and positions of users were periodically updated throughout the simulation. The Euclidean distances were computed from the eNB in the network within the distance matrix. The path losses tested on the signal were predetermined from this distance matrix, as well as the Rayleigh fading and log-normal shading in multipath scenarios. In the network, every eNB updates the OP and PPHP report throughout the simulation. The eNB also updates the load report and sends the load information to other eNBs in the network.

Usually, the HO decision algorithm is made according to the received signal, represented by reference signal reception strength (RSRS) or signal-to-interference-plus-noise-ratio (SINR), as well as the conditions of loading for both the service and target BSs. 

The application of more practical algorithms for the HO decision [68,69] are mathematically provided as follows:

(7) $$RSRS_{s}>\left(RSRS_{t}+HOM\right)$$

where *RSRSs* represents the service BSRS, and *RSRSt* is the target BSRS. The average received signal level was computed from the UE’s perspective across the carrier by every UE and then it moves to the eNB specific to begin the optimisation operation. In addition, the eNB system implements the modulation scheme selection and coding based on the reports of the received signal level [70], and the process of self-improvement is then implemented. Generally, the service eNB provides the HO decision based on the reports of the measurement and estimation of HCPs after completing the self-optimisation process. This is achieved by implementing the HO action sequence in 3GPP, where contact with the UE will be preserved if the eNB service offers satisfactory signal quality [62,71]. 

The simulation model has been applied to the developed algorithm to verify its performance in the system. Then, the results of simulation execution were analysed for the developed algorithm, and the dynamic estimation execution of two HCP settings (HOM and TTT) was compared with various mobile speeds. Throughout the study simulation, six different UE mobile speeds were considered with the following values: {40, 60, 80, 100, 120, and 140} km/h. The effect of various mobile speeds on the performance of the network has been evaluated. The mobile speeds represent the speed properties of vehicles in urban and suburban areas; therefore, they are acceptable in theoretical investigations. As part of the analytical framework, Table 1 presents the values of specific 5G parameters defined in the 3GPP specifications (Rel. 16) that have been taken into account in the simulation.

## 5. Simulation and Performance Evaluation Analysis

This section introduces the combined results of the simulation study and discusses the performance results of the suggested algorithm by comparing the dynamic estimation of two HCPs settings (HOM and TTT). The studied algorithm was examined using six different mobile speed scenarios, HOM levels and TTT intervals to fully illustrate its performance throughout various conditions. This section displays the HO performance for different fixed HCPs to address HPO, based on mobility scenarios. The performance is quantified using the average values calculated across all UEs in the cells over simulation periods with different UE velocities in 5G networks. The effects of various fixed HOM levels and fixed TTT intervals with various UE speeds on system performance have been investigated. The mobility robustness of the 5G network can be verified using different HOM and TTT settings, selected according to 3GPP [15] with the following values: HOM = {0, 2, 4, 6, 8, and 10} dB and TTT = {0, 320, 640, 1280, 2560, and 5120}. To analyse the performance of the proposed algorithm, simulations were successfully conducted with the consideration of various UE speeds. The suggested algorithm was then compared with various optimisation algorithms, such as fixed HOM values and fixed TTT intervals. The overall simulation time for the suggested algorithm was assessed utilising three MPMs: HOP, PPHP and OP. The simulation results for the probabilities of HOP, PPHP and OP were acquired by comparing the performance between fixed HOM values and TTT intervals using the MATLAB simulation software. The key parameters used in this simulation are shown in Table 1.

**Figure 4:**
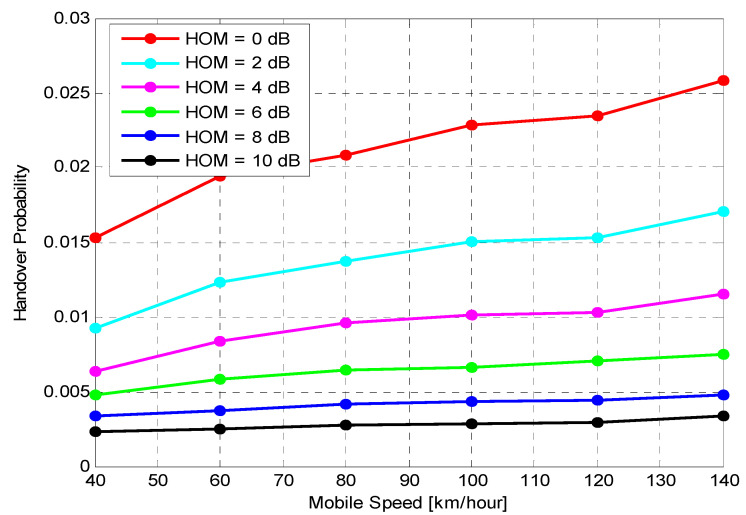
Handover probability versus UE speeds.

**Figure 5:**
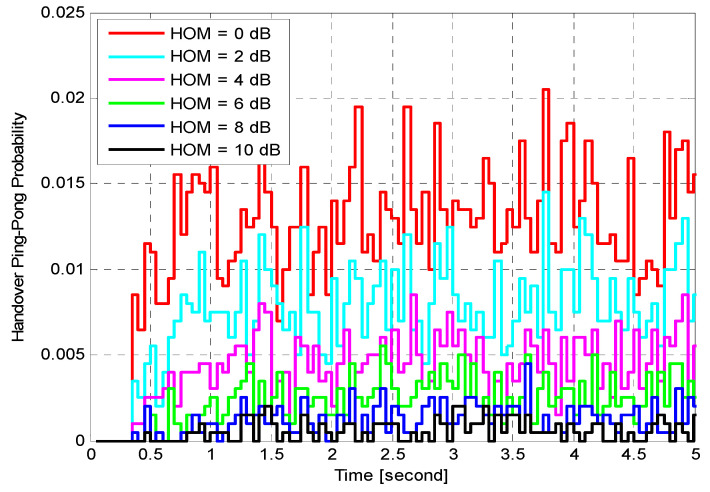
Handover ping-pong probability versus time.

**Figure 6:**
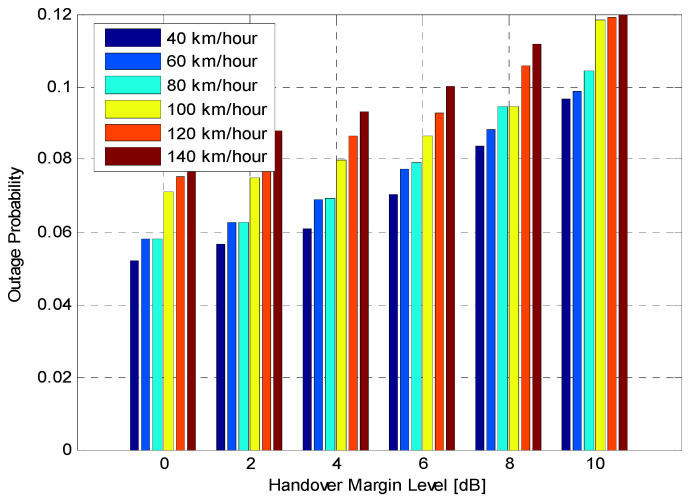
UE outage probability versus HOM level.

**Figure 7:**
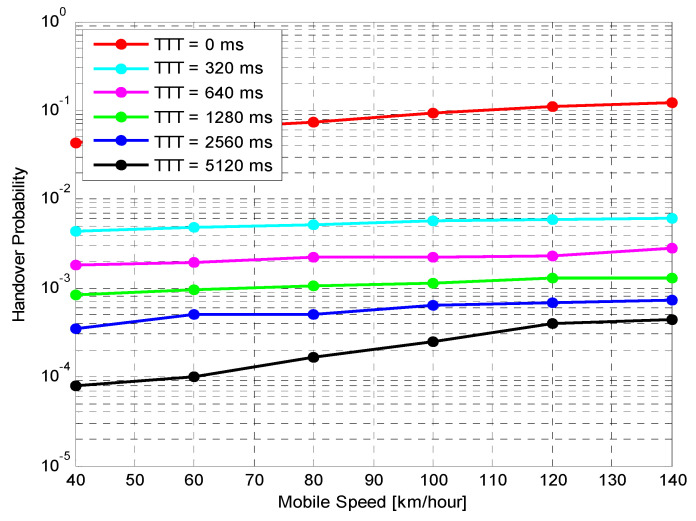
Handover probability versus UE speeds.

**Figure 8:**
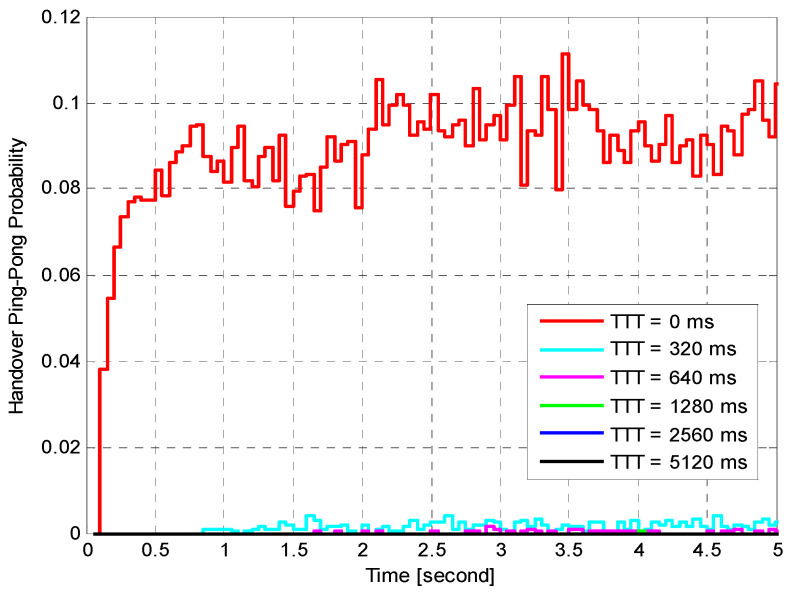
Handover ping-pong probability versus time.

**Figure 9:**
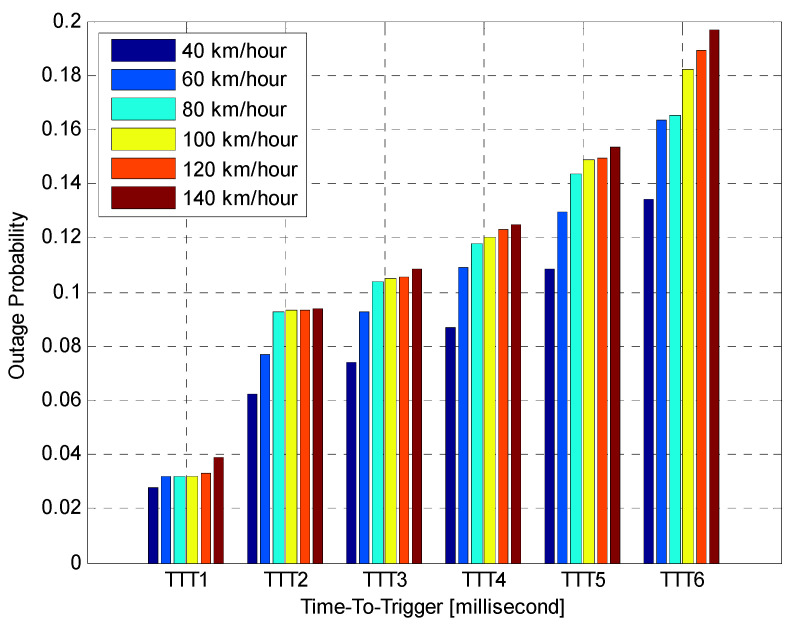
UE outage probability versus TTT interval.

- Performance of Fixed HOM Values
  To analyse the performance of the proposed algorithm with fixed HOM values, simulations with various mobile speeds were performed. Figure 4 presents the average HOP performance of the suggested algorithm for different fixed HOM values under various UE velocities. The suggested algorithm dramatically reduces the average HOP for higher HOM values compared to lower values for all velocities. In this figure, the results indicate that the proposed algorithm with higher HOM (i.e., 10 dB) produces lower HOP, which further increases with time. Nevertheless, with mobile velocity scenarios that are equal to or greater than 60 km/h, the suggested algorithm produced HOPs that were capable of rapidly fluctuating with time for all different HOM values. The outcomes indicate that the suggested algorithm with 10 dB HOM offers remarkable reduction gains in the average HO rate for all mobile speed scenarios. When compared to lower values of HOM (i.e., 0 and 2 dB), the overall average HOP obtained when HOM was 10 dB is 88%. This is 80% less than what was obtained when HOM was 0 dB and 2 dB, respectively. Nevertheless, higher or lower HOP is not always considered as a good or bad indicator. The most important performance indicators are PPHP and OP, as discussed in the following sections.
  Figure 5 displays the impact of HOPP with different HOP values for a specific time. The suggested algorithm for 10 dB HOM obtained lower HOPP rates than other HOMs because of the proper preparation of HCPs and contact with the better eNB target. Nevertheless, the proposed algorithm and other HOM values also acquired low HOPP rates over a specified period, especially in high HOM scenarios where high HOPP rate caused a significant waste of resource mass from the round-trip switching of UE data. The HOPP impact with 10 and 8 dB HOMs at a time of 3.75 s is high for the proposed algorithm compared to other time scenarios. At low speed, the received signals increasingly fluctuate; therefore, the HOPP rate rises. In the middle and high HOMs, the UE connection to the target eNB is faster, resulting in low HOPP rate. The proposed algorithm achieved a significant decrease in the HOPP rate in low HOM scenarios compared to other HOMs. Thus, the proposed algorithm with 10 dB HOM significantly reduces the HOPP rate compared to other HOMs.
  Figure 6 presents the average OP rate of the proposed optimisation algorithm for different HOM levels at varied scenarios of mobile speed. The average OP rate for each HOM level is calculated over all scenarios of mobile phone speed and during the whole simulation time. The OP rate is acquired through the optimisation algorithm, which is significantly reduced for the 40 km/h mobile speed, compared to other mobile speed scenarios. Using an inappropriately modified UE speed for optimising HCPs (i.e., an algorithm based on a mobile phone speed of 140 km/h) may result in high OP rates for all HOM levels. Thus, HCPs must be periodically adapted according to each of the UE’s independent experiences. The impact of the Doppler Effect accompanied by weak connections will further increase the OP rate according to the UE speed. However, at the 6 dB HOM level, the proposed algorithm with 140 km/h mobile speed achieved approximately 8%, 12%, 20%, 22% and 30% average OP rates at 120, 100, 80, 60, and 40 km/h mobile speed scenarios, respectively.
- Performance of Fixed TTT Intervals
  Figure 7 illustrates the average HOP at various UE speed scenarios for different fixed TTT intervals. The performance of the proposed algorithm was compared with different TTT intervals. The simulation results reveal that the proposed algorithm reduces the average HOP for all scenarios of mobile velocity when the TTT is equal to 5120 ms, as compared to other TTT intervals. The total average HOPs achieved through the proposed algorithm are approximately 50%, 60%, 87%, 95% and 99.5% lower than those achieved by 2560 ms, 1280 ms, 640 ms, 320 ms and 0 ms, respectively, for 100 km/h mobile speed scenario. A long HO delay will lead to increased HOP rate for various mobile speed scenarios, since many packet transmissions are disabled through vertical HO. The proposed algorithm achieves a low HOP due to effective HCP values according to UE velocity; therefore, the HO delay is greatly reduced.
  Figure 8 displays the evaluation of the average PPHP rate for the optimisation algorithm, based on different considered values of TTT intervals, as well as the time of the entire simulation. The PPHP rate obtained for the 0 ms TTT interval is relatively higher than other TTT intervals for all simulation time scenarios. This finding is justified since the improper optimisation of HCPs by the MRO algorithm increases PPHP or UHO. High HOP may further lead to an increase PPHP and HOF, while a large decline in HOP will reduce the PPHP rate. The results indicate that in the initial period of operation, PPHPs were low and gradually increased with time. This case is more apparent in the average PPHP over low TTT scenarios, especially for 0 ms TTT. The operation of the network begins based on the HCP settings initially selected; after that, the HCP settings are automatically optimised and updated through the studied algorithm, resulting in various effects on PPHP, which differ according to the reaction and optimisation strength of the algorithm.
  In Figure 9, the outcomes reveal that the average OP recorded throughout all measured utilizers and scenarios of mobile speed are based on the various TTT intervals that have been taken into account. In this figure, the OPs are served as the average rate for all measured utilizers with different TTT interval scenarios. In general, the results demonstrate that the suggested algorithm continuously interacts with time, and OPs are subject to change over time for all scenarios of mobile speed. However, in Figure 6, the suggested algorithm with a 40 km/h mobile speed presented a remarkable reduction in the OP rate in comparison with other selected speeds for all TTT intervals. The proposed optimisation algorithm with 140 km/h mobile speed scenario basically caused the highest OP rate as the average over all different TTT interval scenarios. The average reduction gains achieved through the proposed optimisation algorithm with 40 km/h mobile speed scenario and TTT were approximately 32%, 29%, 26%, 17% and 16% lower for 140, 120, 100, 80 and 60 km/h mobile speed scenarios, respectively. This represents a significant achievement for the application of the proposed optimisation algorithm.
  The simulation results reveal that the performance of the algorithm with fixed HOM values outperforms the performance of the algorithm with fixed TTT intervals throughout all performance metrics with the scenarios of various speed. In addition, the HOP and PPHP behaviours with different HOM and TTT values correspond to the impact of HOM on both parameters. At low TTT intervals, the HOP and HOPP rates increase and raise the level of signals, while at high TTT periods, both rates reduce and decrease the levels of signals. The high velocity of UE maybe cause OP to rise, since the serving BS or the target BS is not servicing the UE.
  In general, this study was performed based on a simulation study only. To the best of our knowledge, most handover management studies are conducted based on simulations. Moreover, it has become very difficult to acquire data from the operator regarding mobility management. Most of the data we received are not useful and cannot be used to study handover management. The acquired data do not consider the handover control parameter settings and it is also not clear which handover decision algorithm was used.

## 6. Conclusions

This paper verified the effect of different HCP settings on 5G network performance by analysing fixed HCP settings in various scenarios to explain the need for applying more advanced technology in 5G networks. The proposed algorithm in this study was used to assess the HCP settings. These optimisation values were estimated according to the speed of UE’s and the loads of cell. Moreover, the suggested algorithm provides liberty to the service network through independently setting HCP values for all UEs; therefore, all UEs acquire varied HCP settings from other UEs. The MRO adjusts HCPs (such as the HOM and TTT) to maintain connection links throughout user mobility with minimum operator interference. The effects of different fixed HOM values and fixed TTT intervals on system performance at various mobile velocities were examined. The proposed algorithm monitors UE speed through UE mobility and then determines the appropriate HOM and TTT values to meet all requirements for successfully implementing the HO process. The proposed system scenarios were evaluated by analysing various HCP settings. A comparison of three MPMs with different mobile speeds was also included: HOP, PPHP and OP. The influence of utilizer mobility on the performance of system was further investigated, where the average experienced HOP was obtained over various HOM and TTT settings across different scenarios of mobile speed and during different time periods in the 5G network. The simulation results show that the performance of the proposed system provides noticeable improvements for OP with the use of lower HCP settings, as compared to higher HCP settings. However, a noticeable drawback can be seen in terms of increased PPHP for different mobile speed scenarios. These results indicate that medium HCP settings may be the best solution when using one of these proposed systems. The simulation results also revealed that the average rates of PPHPs and OP with fixed HOM values are significantly reduced by the proposed algorithm, compared to that of fixed TTT intervals. For further investigation and development, automatic optimisation should be implemented.

In addition, mobile speed has been considered as a maximum of 140 km/hr, to reflect the real environment for vehicle speeds. To the best of our knowledge, and based on our observations with recommendations from professors, the average maximum movement speed for a car is suggested to be around 140 km/h in a study of this type. Moreover, based on the test drive study conducted in urban areas, on suburban highways, and rural areas [72,73] for a project we performed, the recommended movement speed for the car was 60 km/h to facilitate reliable measurements. Furthermore, we are currently working on different mobile speed scenarios that consider train speed and drones, which will be up to 500 km/hr. The results of these new scenarios will be published in the next research paper.

## Figures and Tables

**Table 1:** Summary of the HO optimisation approaches.

Ref.	Description	Decision Making Method	Wireless Network Type	Given Parameters	Pros and Cons
[55]	An algorithm has been suggested to reduce the HOs by optimized MCDM algorithms with a context-aware and threshold-based scheme.	TOPSIS, PROMETHEE, and SAW	5G-Ultra Dense Network (UDN)	Average number of Hos, Euclidean average distance.	Significantly reduces the number of UHOs and improves QoS.
[7]	The algorithm of velocity-based self-optimization was suggested to modify the values of HCP according to the velocity of the UE and the RSRP. The suggested algorithm has proven to be effective under various mobile velocities, compared to other existing algorithms.	Velocity-based self-optimization	4G and 5G	HOP, PPHP, and RLF	A noticeable decrease according to the total probability rate for RLF, HOP, and HOPP.
[56]	A mathematical model of CRAT was derived by taking into account the context of the user and the network, by adopting an analytic hierarchical process (AHP) to weight the importance of selection criteria and TOPSIS for classifying the available RATs.	AHP and TOPSIS	5G-UDN	Number of HOs, average network delay, packet delivery ratio and throughput.	The proposed CRAT outperforms the traditional A2A4 approach to the selection of RAT.
[49]	The maximum likelihood (ML) estimator for speed estimation has been proposed in HetNets by separately utilizing sojourn time and HO count measurements. Velocity estimate based on sojourn time is more accurate than HO number because it uses both sojourn time and HO count information.	Sojourn time and HO count	HetNets	Root-mean-square error (RMSE), total number of HO, HOF and UHO, mobility state probabilities, probability of detection, and probability of false alarm.	Accuracy in estimating the speed, limit frequent HOs and the failure of the service, and mobility state detection (MSD) optimization.
[57]	Fuzzy Q-Learning was used to improve the two contrasting HO problems, namely ping-pongs and RLFs through adjusting the HOM and TTT.	Fuzzy Q-Learning	LTE	PPHP and RLF	The suggested algorithm is robust against changes in the users number in the system, where it keeps the better solution when the users number is halved or doubled.
[58]	A distributed MRO algorithm was proposed to improve the performance of HO by minimising RLFs, where the proposed algorithm classifies HOFs based on the causes of failure.	Adjust TTT and offset parameters	LTE	RLF rates and ping-pong rates	Adaptively optimizes the HO parameters, stimulates the least number of ping-pongs among the algorithms studied, and thus outperforms the previous algorithms.

**Table 2:** The system simulation parameters [62,67].

Parameters of Network	Presumption
Environment	5G Rel. 16 System, micro cells and urban areas
Hexagonal Cells No.	Changes dynamically according to the simulation time
Number of sectors for each cell	3
Height of eNBs antenna	15 m
R (m)	200 m
System bandwidth	500 MHz
Total power TX eNB	46 dBm
Shadowing	8 dB
Tested UE number	15 UEs randomly distributed
Noise Figure of UE	9 dB
eNB noise figure	5 dB
Height of UEs	1.5 m
No. and type of UE antenna	1, Omni-directional
No. of users inside each hexagonal cell	200
Simulation cycle	5 s
Mobility Model	Directional
The speeds of UEs	{40, 60, 80, 100, 120, 140} km/h
Min. desired level of RX in the cell [3GPP TS 36.304]	−101.5 dBm
Initial value of HOM	2 dB
Initial value of TTT	100 ms
Min. and max. HOM values	(0 and 10) dB
TTT intervals	Changes from (0 to 5.12) s

## Data Availability

Not applicable.

## Abbreviations

The following acronyms are used in this manuscript.

**Acronyms**			
3GPP	3rd Generation Partnership Project	MR	Measurement Report
4G	Fourth Generation	MRO	Mobility Robustness Optimisation
5G	Fifth Generation	NCL	Neighbouring Cell List
ASO	Automatic Self-Optimisation	NSA	Non-Standalone
AWGN	Additive White Gaussian Noise	OP	Outage Probability
BS	Base Station	OFDMA	Orthogonal Frequency Division Multiple Access
CRAT	Context-aware Radio Access Technology	PPHP	Ping-Pong Handover Probability
eNB	Evolved Node B	PROMETHEE	Preference Ranking Organization Method for Enrichment Evaluation
HCP	Handover Control Parameter	QoS	Quality of Service
HO	Handover	RFA	Reverse Frequency Allocation
HOF	Handover Failure	RATs	Radio Access Technologies
HOM	Handover Margin	RRC	Radio Resource Control
HOP	Handover Probability	RT	Residence Time
HPO	Handover Parameter Optimisation	SA	Standalone
ICI	Inter-Cell Interference	sBS	small cell BS
IoTs	Internet of Things	SIN	Self-Improvement Network
KPIs	Key Performance Indicators	SINR	Signal to Interference plus Noise Ratio
LB	Load Balancing	SON	Self-Improvement Networks
LBEF	LB Efficiency Factor	ST	Stay Time
LBHSO	LB Handover Self-Optimisation	SAW	Simple Additive Weighting
LBO	Load Balancing Optimisation	TOPSIS	Technique for Order Preferences by Similarity to the Ideal Solution
mBS	macro-cell BS	TTT	Time To Trigger
MCDM	Multi-Criteria Decision-Making	UE	User Equipment
MLB	Mobility Load Balancing	UMLB	Utility-based MLB
MPCs	Major Performance Criteria	URC	Ultra-Reliable Communication
**Notations**			
$\bar{HOP}$	The HOs average number per UE		
$M_{UE_{s}}$	The total number of served UEs in the whole simulation through the network		
HOP(i)	The HOP for UEi		
$T_{l}$	The time that UE leaves the serving eNB-A		
$T_{r}$	The time that UE returns back to the same serving eNB-A		
$T_{c}$	The critical period		
$\bar{PPHP}$	The average HPPP for UE through the simulation period 𝑡		
$M_{PPH}$	The total number of PPH throughout the entire system		
$M_{F}$	The total number of requested HOs represents the sum of failed HOs		
$M_{NPPH}$	The total number of non-PPH		
η	The immediately received SINR level		
*η*th	The threshold level is the minimum SINR level		
$\bar{OP}$	The average OP		
$M_{UE_{s}}$	The total number for all UEs		
$H_{\max}\;$ and $H_{\min}$	The maximum and minimum HOM values		
$M_{ap}$	The adjusted part of HOM.		
RSRSs	The service BSRS		
RSRSt	The target BSRS		
